# Building an Adaptable Pediatric Intensive Care Unit Simulation Portfolio: Advancing Efficiency, Flexibility, and Team-based Training

**DOI:** 10.1097/pq9.0000000000000864

**Published:** 2025-12-23

**Authors:** Daniel Loeb, Kelly Ely, Kelly Collins, Zachary Paff, Corey Eyer, Maya Dewan, Matthew W. Zackoff

**Affiliations:** From the * University of Cincinnati College of Medicine, Cincinnati, Ohio; † Division of Critical Care, Department of Pediatrics, Cincinnati Children’s Hospital Medical Center, Cincinnati, Ohio; ‡ Center for Simulation and Research, Cincinnati Children’s Hospital Medical Center, Cincinnati, Ohio; § James M. Anderson Center for Health Systems Excellence, Cincinnati Children’s Hospital Medical Center, University of Cincinnati College of Medicine, Cincinnati, Ohio.

## Abstract

**Introduction:**

Simulation-based education enhances patient safety and team performance in high-acuity environments, such as the pediatric intensive care unit (PICU). However, many units struggle to implement scalable, dynamic simulation programs adaptable to fluctuations in patient volume, clinical acuity, and staffing demands. This quality improvement initiative aimed to develop and implement a comprehensive PICU simulation portfolio, integrating predictive analytics, multidisciplinary collaboration, and real-time clinical data to increase the frequency of simulations and training exposures.

**Methods:**

The PICU simulation portfolio underwent iterative improvement via a structured quality improvement initiative. Guided by a specific, measurable, achievable, relevant, and time-bound aim and key driver diagram, targeted interventions were implemented through iterative plan-do-study-act cycles. These interventions included introducing standardized simulation encounter types, a simulation encounter decision tree informed by real-time predictive analytics, incorporating a multidisciplinary team, centralizing simulation resources, and streamlining the setup process. Primary outcomes included simulation frequency and cancelations. Secondary outcomes included participation, facilitator workload, and safety event monitoring.

**Results:**

Over the intervention period (July 2022 through June 2024), the simulation program demonstrated increased training capacity, improved efficiency, and heightened participation. Annual simulations increased by 128%, incorporating 1,256 staff, whereas cancelations decreased by 25%. Simulation setup and takedown time were reduced by 58% and 64%, respectively. There were no reported safety events.

**Conclusions:**

This scalable PICU simulation training model led to measurable gains in efficiency, training capacity, and multidisciplinary collaboration. Its adaptable framework offers a promising approach for other clinical settings aiming to enhance simulation-based education and training to improve patient care outcomes.

## INTRODUCTION

Simulation-based training is essential in medical education,^1–3^ offering a safe environment to build clinical skills, teamwork, and confidence.^4–7^ By providing a low-risk environment for healthcare professionals to practice and refine their skills,^3^ simulation training improves clinical competencies,^1,4,7^ fosters effective team dynamics, and enhances patient safety. It allows practitioners to rehearse complex procedures, manage rare or critical events, and internalize best practices without compromising patient care.^7,8^ Numerous studies have demonstrated that simulation boosts individual confidence and proficiency,^9^ leading to better outcomes and fewer errors.^10,11^

Simulation-based training is especially relevant in critical care, where high patient acuity and the potential for rapid deterioration demand coordinated team responses. In situ simulation builds skills for managing life-threatening events while improving communication, collaboration, and clinical readiness.^5,12,13^ As such, the pediatric intensive care unit (PICU) at Cincinnati Children’s Hospital Medical Center (CCHMC) has maintained a simulation program for several years, consisting of both in situ (ie, within the actual clinical environment) and off-site laboratory-based simulation sessions encompassing both individual clinician roles and interprofessional teams.

Despite clear benefits, scaling in situ simulation in high-acuity settings remains challenging.^14^ Multidisciplinary participation is hard to sustain, training must adapt to evolving clinical needs, and fluctuating staffing can disrupt sessions. In high-stress settings, staff often work near capacity, limiting their ability to engage in simulation-based learning.^15^ Stated another way, the cognitive and emotional resources essential for effective learning are often insufficient, limiting the ability of staff to fully benefit from training sessions.^16^ Simulation programs in high-acuity settings must therefore cultivate adaptive capacity—defined as a system’s ability to reconfigure its structures and functions in response to shifting clinical conditions—so that performance remains reliable despite constant change.^17^ In July 2022, the CCHMC PICU launched a quality improvement initiative with a specific, measurable, achievable, relevant, and time-bound (SMART) aim to expand simulation capacity and reliability within 2 years.

## METHODS

### Context, Setting, and Study Period

This quality improvement initiative was conducted in a 48-bed closed PICU at a single quaternary pediatric referral center with more than 2,500 annual admissions. The unit serves as a major transplant and level 1 trauma center, managing complex and rare cases. Data were collected from July 1, 2022, to June 30, 2025. The project received institutional review board exempt status due to its quality improvement focus.

### Study Measures

The SMART aim was to increase the frequency and reliability of the PICU simulation program as measured by an increase in annual simulations by 50% (from a baseline of 39 to a target of 59 sessions) and a reduction in cancelations by 25% (from 19% to 14%) between July 1, 2022, and June 30, 2024. Primary outcome measures included the number of scheduled, conducted, and canceled simulation sessions. These outcomes reflect the simulation program’s ability to function reliably within a high-acuity environment. Cancelations, although not direct proxies for educational quality, hinder consistency and sustainability. Tracking them helped assess program reliability under clinical pressure.^14^ The number of executed simulations represented opportunities for experiential learning and skill reinforcement. Secondary measures included simulation type, participant roles, timing (on- versus off-shift), and location (in- versus outside the PICU), as summarized in Table 1. Simulation team time per session was tracked as a process measure, and workflow disruptions served as the balancing measure.

**Table 1.** PICU Simulation Courses Scheduled and Ran from July 2021 to June 2025

Course	July 2021–June 2022		July 2022–June 2023		July 2023–June 2024		July 2024–June 2025	
	Scheduled	Ran	Scheduled	Ran	Scheduled	Ran	Scheduled	Ran
Simulations occurring during clinical shifts in the ICU (on-shift)								
In situ Simulation	16	12	22	17	—	—	—	—
Core Team Training*	—	—	—	—	11	7	13	12
ED to PICU eCPR*	—	—	—	—	5	5	6	6
Just-in-Time Training*	—	—	—	—	16	13	13	10
Rolling Refresher*	—	—	—	—	18	13	20	17
Subtotals	16	12 (75%)	22	17 (77%)	50	38 (76%)	52	45 (86%)
Simulations occurring outside of clinical shifts in the ICU								
Multidisciplinary eCPR Simulation (Surgery/ECMO)	4	3	4	4	5	5	5	5
House-wide Codes Day	—	—	2	1	6	6	7	6
Nursing Orientation	14	14	18	18	14	14	6	6
Experienced Nursing Seminar	—	—	—	—	5	5	12	12
Fellow Conference Simulation	—	—	—	—	4	2	6	5
Fellow Procedural Training Boosters	2	2	4	4	3	3	4	3
Advanced Practitioner CPR Coach Curriculum	—	—	—	—	—	—	33	33
Subtotals	20	19 (95%)	28	27 (96%)	37	36 (97%)	67	64 (96%)
Simulations occurring outside of clinical shifts and outside of the ICU (off-shift)								
PICU Team Safety	8	5	12	10	12	11	12	12
Fellow Boot camp	2	2	2	2	2	2	2	2
Fellow Advanced Ultrasound Workshops	2	1	2	2	2	2	2	2
Sub—totals	12	8 (67%)	16	14 (88%)	16	15 (95%)	16	16 (100%)
Program totals	48	39 (81%)	66	58 (88%)	103	89 (86%)	135	125 (93%)

Courses marked with an asterisk

(* ) were subdifferentiated from the more broad “In-situ Simulation” course, which was retired before the start of the 2023 academic year. Results noted by an (—) indicate that the course was not in existence that year.

CPR, cardiopulmonary resuscitation; ECMO, extracorporeal membrane oxygenation; eCPR, extracorporeal cardiopulmonary resusciation.

### Planning and Studying the Interventions

**Fig. 1.**
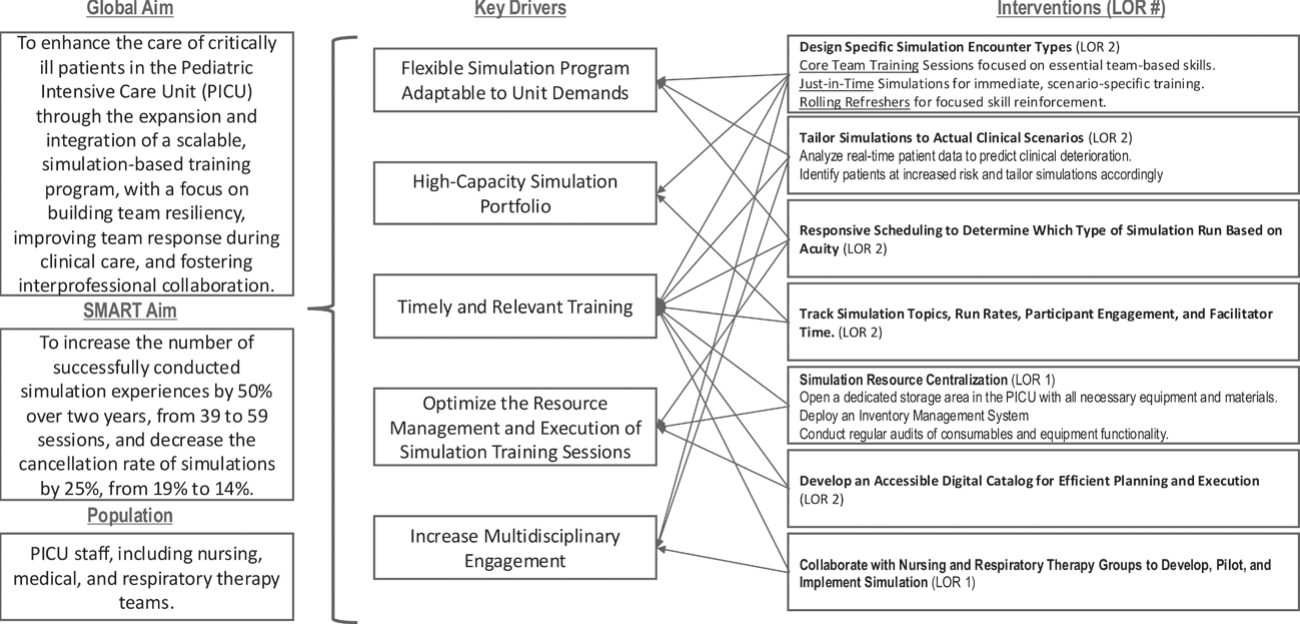
Key driver diagram outlining the global and smart aims, key drivers, and interventions for the quality improvement initiative. LOR, level of reliability.

The team included 2 pediatric critical care attendings with simulation expertise, 1 with quality improvement expertise, a PICU Advanced Practice Registered Nurse, 2 PICU nurses, and a PICU respiratory therapist, all with simulation experience. This interprofessional group developed a key driver diagram outlining aims, key drivers (flexible simulation program adaptable to unit demands, high-capacity simulation portfolio, timely and relevant training, optimized resource management and execution of simulation training sessions, and increased multidisciplinary engagement), and interventions (Fig. 1). Using the Model for Improvement in Healthcare, the team conducted multiple plan-do-study-act (PDSA) cycles.^18^ The team monitored outcome, process, and balancing measures throughout the study. It mapped each intervention to systems engineering initiative for patient safety (SEIPS) 2.0 domains to illustrate system-level changes,^19^ highlighting the structural changes that enabled more frequent, reliable simulation delivery. (**See table 1, Supplemental Digital Content 1**, which displays the PDSA cycle mapped to the SEIPS work system elements. SEIPS 2.0 elements include people, tasks, tools/technology, physical environment, and organization/policies, https://links.lww.com/PQ9/A735.) This quality improvement initiative was mapped to the standards for quality improvement performing excellence-simulaton guidelines^20^ for simulation-based quality improvement studies.

Primary outcomes—scheduled, ran, and canceled simulations—were tracked through an institutional simulation tracking platform in place before 2021. To capture secondary outcomes and process measures, a PICU simulation session tracking system was implemented on July 1, 2023. This system recorded encounter type, clinical topic, and participant roles. Perceived simulation-associated workflow disruptions, a key balancing measure, were monitored longitudinally through passive surveillance of the voluntary institutional safety event recording system.

#### PDSA Cycle 1: Interprofessional Collaboration and Participation

To expand the simulation program’s reach and reliability, starting in July 2022, we prioritized interprofessional collaboration to improve simulation quality and foster unit-wide buy-in. Nursing, medical, and respiratory therapy leaders helped refine scenario-specific learning objectives. Interested staff members received additional training in simulation setup and design and assisted with execution as part of their professional development. (**See figure 4, Supplemental Digital Content 2**, which displays the PICU simulation program work system network. The number of unique participants in each node is denoted in each cell, https://links.lww.com/PQ9/A733.) In March 2023, a 0.5 full-time equivalent nursing education leader—a PICU nurse with acute care certification and deep critical care expertise—was hired to support simulation development, execution, and operational improvements to increase efficiency (defined in this project as reducing the time, labor, and operational friction required to plan, set up, run, and break down simulations).

#### PDSA Cycle 2: Standardization of Simulation Encounter Types

To ensure consistency in simulation training, we established distinct encounter types with standardized goals, required equipment, involved staff, and session durations. Simulations were categorized as in situ (within the PICU) or laboratory-based (off-site). We further divided on-unit simulations into sessions involving on-shift staff or prescheduled staff outside of clinical shifts. All laboratory-based sessions involved staff outside of clinical shifts, and nursing and respiratory therapists received credit toward paid education hours for participating. With regard to educational goals, the simulations “outside of clinical shifts” that occur either in the intensive care unit (ICU) or outside the ICU constitute the core training curricula for PICU staff. Participant-level tracking ensures all staff participate in these sessions and that any content deemed a core component of training is embedded in them. Meanwhile, simulations occurring “during clinical shifts” supplement the core curriculum by focusing on recent events and adapting to the unit’s current acuity and complexity. Learnings from these sessions are then incorporated into the core off-shift simulations to ensure all staff receive necessary training. As such, there is no individual participant-level tracking of participation in simulations “during clinical shifts.”

In July 2023, we segmented on-shift in situ simulations into 3 types: Core Team Training, Just-in-Time Simulations, and Rolling Refreshers. Core Team Training focuses on unit-wide events, such as code responses, emphasizing interprofessional communication and clinical reasoning. These sessions may or may not mirror real patients and require participation from all interdisciplinary PICU team members. Just-in-Time Simulations are focused, context-specific sessions that reflect real patients in near–real time, allowing staff to rehearse mitigation plans just before high-risk events. These plans may include staff not directly responsible at the bedside, such as the code response team. These simulations are built and implemented with the same fidelity and realism as Core Team Trainings and similarly require all relevant interdisciplinary team members. Rolling Refreshers are brief, low-fidelity drills with focused objectives, conducted quickly throughout the unit and adapted to the roles of available team members (Table 2). Low-fidelity simulation uses simplified or part-task trainers with limited realism, whereas high-fidelity simulation uses highly realistic, immersive manikins or environments that closely replicate clinical conditions^21^

**Table 2.** Simulation Encounter Types and Specific Courses Within the Comprehensive PICU Simulation Program

Course	Description	Location	Participants	Average No. Participants per Session (July 2023–June 2024)
In situ simulations occurring during clinical shifts (on-shift)				
In situ	Training that takes place on the unit with the staff working at that time in real time This simulation course was retired in 2023 and replaced with “Core Team Training,” “Just-in-Time Simulation,” and Rolling Refreshers	PICU	Respiratory therapists, nurses, advance practice providers, pharmacists, physicians	Retired
Core Team Training	In situ training that takes place on the unit with the staff working at that time. The simulation typically features a cardiac arrest and a complete code team response	PICU	Respiratory therapists, nurses, advance practice providers, pharmacists, physicians	23.9
ED to PICU eCPR in situ	A subset of Core Team Training, these simulations focus on the transfer and management of patients requiring eCPR from the ED to the PICU	ED and PICU	Respiratory therapists, nurses, advance practice providers, pharmacists, physicians	25.5
Just-in-Time	A high-fidelity, clinically integrated simulation conducted in direct response to a real-time patient condition or risk, typically within minutes to hours of clinical care. These simulations replicate actual patients with a high degree of fidelity and realism, and are designed to rehearse anticipated clinical scenarios, reinforce mitigation strategies, and align with current patient deterioration indicators and mitigation plans	PICU	Respiratory therapists, nurses, advanced practice providers, pharmacists, physicians	20.5
Rolling Refresher	Low-fidelity simulations to focus on narrow learning objectives informed by current patient pathology in the PICU	PICU	Respiratory therapists, nurses, advanced practice providers, pharmacists, physicians	13.5
Simulations within the PICU occurring outside of clinical shifts (off-shift)				
Multidisciplinary eCPR Simulation (PICU, Surgery, ECMO Teams)	Exercise focusing on the activation and cannulation of a patient who requires initiation of ECMO during CPR	PICU	Respiratory therapists, nurses, advanced practice providers, pharmacists, physicians	31.4
House-wide Codes Day	A dedicated day for practicing code scenarios, where healthcare professionals simulate emergency responses to cardiac or respiratory arrest to improve teamwork, review roles, and improve response times in the PICU and house-wide	PICU	Respiratory therapists, nurses, advanced practice providers, physicians	11.4
Nursing orientation	Simulation-based orientation and training for new graduate nurses or experienced nurses, transitioning to the PICU who were trained in a different area of nursing, to transition into clinical practice effectively	PICU	Nurses	7.6
Experienced Nursing Seminar	A training day tailored for experienced nurses to professionally grow their clinical skills and critical thinking, through simulation scenarios relevant to their expertise	PICU	Nurses	10.0
Fellow Conference Simulation	A series of lectures for medical fellows covering core topics, often supplemented with simulation exercises to reinforce learning	PICU	Physicians, advanced practice providers	16.0
Fellow Procedural Training Booster Sessions	An individualized curriculum informed by aggregated real-time feedback on bedside performance of critical skills, including intubation, chest tube placement, central venous catheter insertion, and arterial line placement	PICU	Physicians	7.3
Advanced Practitioner CPR Coach Curriculum	Simulation training focusing on high quality CPR coaching and team dynamics required for effective resuscitation	PICU	Advanced practice providers, nurses	Launched in July 2025
Laboratory-based off-site simulations occurring outside of clinical shifts				
PICU Team Safety	Simulation training focused on improving team communication, situational awareness, and safety protocols to reduce errors in clinical settings	Simulation center	Respiratory therapists, nurses, advanced practice providers, physicians	11.5
Fellow Boot Camp	An intensive training program for medical fellows, providing hands-on simulation experiences to prepare them for advanced responsibilities	Simulation center, PICU	Physicians	22.5
Fellow Advanced Ultrasound Workshops	Specialized workshops for fellows to develop advanced skills in ultrasound techniques through hands-on simulations	PICU	Physicians	14.5

CPR, cardiopulmonary resuscitation; ECMO, extracorporeal membrane oxygenation; eCPR, extracorporeal cardiopulmonary resusciation; ED, emergency department.

To align in situ on-shift simulations with clinical needs, we developed related scenarios across all 3 encounter types. For example, a high-risk tension pneumothorax case informed: (1) a Core Team Training on tamponade physiology and cardiac arrest, (2) a Just-in-Time Simulation on recognizing vital sign changes and performing needle decompression, and (3) a Rolling Refresher covering tamponade physiology and bedside mitigation strategies.

#### PDSA Cycle 3: Simulation Encounter Type Decision Tree

To guide selection of the most appropriate simulation encounter type (PDSA 1) for the dynamic PICU environment, the team implemented a decision tree based on real-time unit characteristics and acuity. It incorporated objective indicators—patient census, staffing ratios, and high-risk flags from the PICU Warning Tool—to align simulation capacity with current conditions. (**See figure 1, Supplemental Digital Content 3**, which displays the unit-driven in situ simulation scaffold. A simulation encounter decision tree allowed for consensus between the simulation team and the clinical staff to help determine the most appropriate type of simulation that could be run based on unit status, https://links.lww.com/PQ9/A724.) Operational leaders applied “no-go” criteria (eg, understaffing, multiple resuscitations, surge status) during planning huddles to avoid care disruption. These criteria applied to both on- and off-shift simulations in the ICU, as off-shift sessions still occupied active patient rooms and were prone to late cancelations.

PICU operational and clinical leaders collaborated with the simulation team to ensure the decision tree prioritized patient safety, minimized distractions, and prevented staff overload that could compromise educational goals. The tool shifted the conversation from *whether* a simulation should occur to *what type* should occur. For example, during low-acuity periods, stakeholders agreed that a Core Team Training—often involving a unit-wide code response and deeper reflection—could proceed. In high-acuity or high-volume situations, focused formats such as Just-in-Time or Rolling Refreshers were prioritized to reinforce critical skills without disrupting unit workflow.

During the development of the simulation encounter decision tree, the team collaborated with PICU operational and clinical leaders to identify times that minimized care disruption, maximized participation, and avoided overtaxing staff. Preferred windows included: (1) between morning rounds and midday conferences/lunch, (2) early afternoon before anticipated admissions, and (3) evening after shift change but before the midnight safety huddle. We implemented the decision tree in November 2023. Simulations were not scheduled on a fixed calendar but strategically deployed based on these time windows and real-time unit conditions. Cancelation and completion rates are shown in Table 1.

#### PDSA Cycle 4: Leveraging Predictive Analytics and a Structured Scenario Selection Workflow to Increase Timely and Relevant Training

After establishing an interprofessional team (PDSA cycle 1), standardizing simulation types (cycle 2), and reaching consensus on simulation selection based on unit conditions (cycle 3), the next PDSA cycle in January 2024 focused on increasing staff buy-in by prioritizing timely and relevant training. By integrating simulation with predictive analytics, the simulation team aimed to align training with real-time needs to enhance preparedness by focusing on scenarios pertinent to patients at the highest risk of clinical deterioration on a given shift.

Our PICU uses a situational awareness system to reduce cardiac arrests, anchored by the PICU Warning Tool—a decision-support tool that flags high-risk patients in real time.^22^ Once flagged, the medical team must develop a patient-specific plan with the bedside staff. Leveraging this structure, the team developed a scenario selection workflow to prioritize timely, relevant training. Simulations were typically based on actively flagged patients, or if none were identified, on recent safety events or important foundational clinical states that had not been covered in recent months. This ensured training remained clinically relevant and responsive to evolving needs. This approach strengthened contextual fidelity and staff participation, while supporting long-term sustainability.

#### PDSA Cycle 5: Programmatic Overhaul of Simulation Organization and Resource Management

Although earlier PDSA cycles improved simulation delivery, efficiency challenges persisted, leaving simulations vulnerable to cancelation during sudden changes in unit status. The next PDSA cycle aimed to improve consistency and reliability by comprehensively reorganizing simulation resources and workflows—consolidating materials, standardizing processes, and reducing friction points that had hindered efficient execution.

**Fig. 2.**
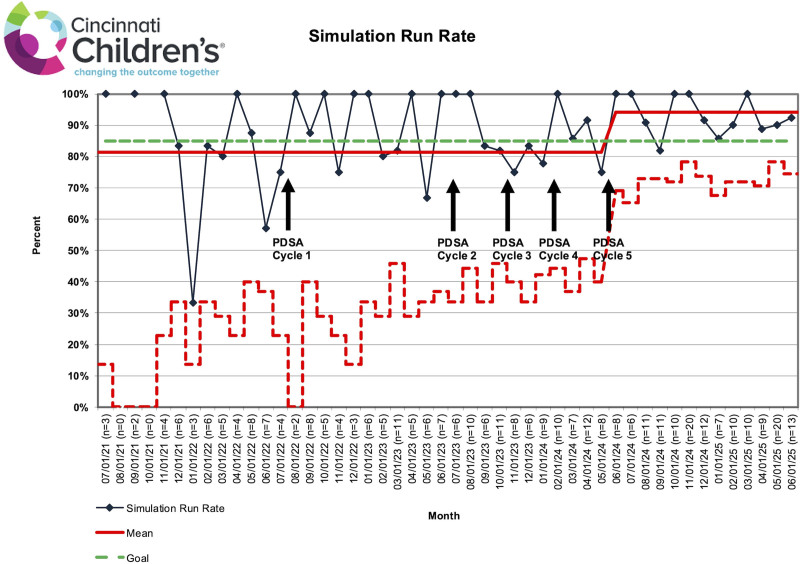
A P-chart demonstrating a stable simulation run rate, defined as the number of simulations conducted divided by the number scheduled per month, throughout the baseline and study periods.

**Fig. 3.**
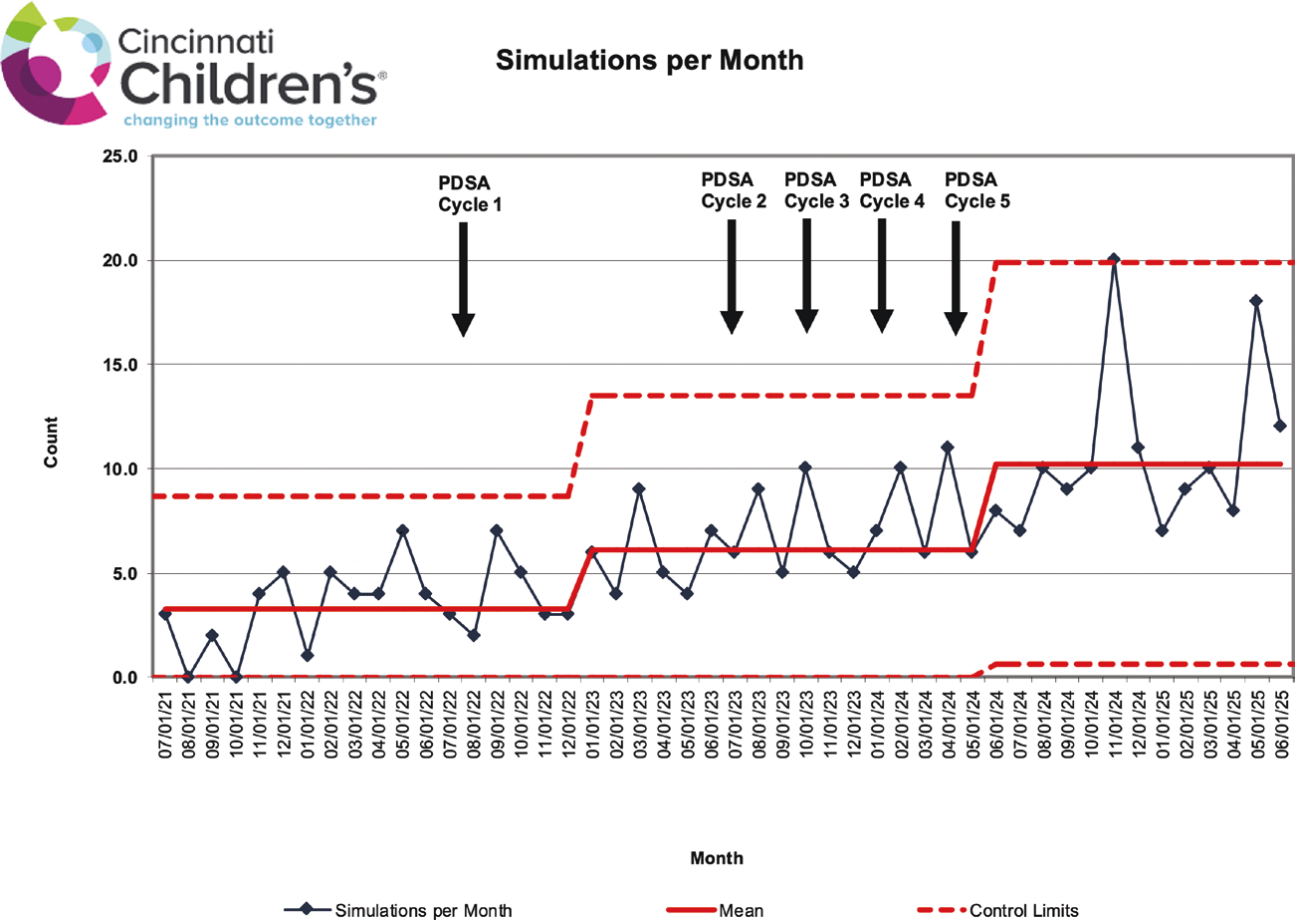
A C-chart demonstrating an improvement in simulations conducted per month during the study period.

A dedicated PICU storage area was established in April 2024 to house standardized “scenario kits,” each containing essential simulation supplies—medical equipment, simulated medications, documentation forms, and ancillary items—and referencing the location of larger core equipment (eg, manikins, defibrillators, code carts). (**See figures 2, Supplemental Digital Content 4**, which displays simulation kits. An example checklist for high-yield items required for simulation, stratified by patient weight and topic, https://links.lww.com/PQ9/A731.) (**See figure 3, Supplemental Digital Content 4**, which displays simulation kits and an example kit checklist. A simulation kit consists of preprepared equipment necessary for content-specific simulations, https://links.lww.com/PQ9/A732.) Kits were assembled by the simulation team in collaboration with interested PICU staff, funded through internal support from the Division of Critical Care, the PICU operational budget, and the central simulation center. Multiple scenarios shared kits, promoting flexibility and reducing redundancy.

Finally, all PICU simulations were standardized into digital templates and were accessible to the simulation team (**See figure 5, Supplemental Digital Content 5**, which displays the example simulation flow diagram. The simulation diagram was used to catalogue, describe, and execute simulations, https://links.lww.com/PQ9/A734.) This catalog included learning objectives, scenario flow diagrams, equipment lists, setup instructions, and debriefing guides centered on the PEARLS (Promoting Excellence and Reflective Learning in Simulation) debriefing framework.^23^

### Data Analysis

Data on scheduled, completed, and canceled simulations were obtained from the institution’s centralized simulation scheduling platform. Additional details—encounter type, clinical topic, participant roles and counts, and simulation team time—were collected via the PICU simulation tracking system. We reviewed the institutional safety event reporting system monthly to identify simulation-associated safety events as a balancing measure.

Primary outcomes (scheduled, completed, and canceled simulations) were tracked using statistical process control charts: a P-chart for simulation run rate, defined as the number of simulations conducted divided by the number scheduled per month, and a C-chart for average simulations per month, with centerlines adjusted per standard statistical process control methods. Descriptive statistics were used to characterize secondary outcomes (simulation types, participant roles, and counts), the process measure (facilitator work hours), and the balancing measure (simulation-related safety events).

## RESULTS

### Primary Outcome Measures

During the intervention period—July 2022 through June 2024—we achieved our SMART aim of increasing the frequency and consistency of the PICU simulation program with a 128% increase in the number of simulations per year from 39 (2021–2022 academic year baseline) to 89 (2023–2024 academic year). During the same period, the simulation session cancelation rate dropped 38%, from 19% (2021–2022) to 14% (2023–2024), meeting the 25% reduction target. With ongoing tracking from July 2024 to June 2025, we sustained these improvements and saw a further increase in our number of simulations per year to 125 (a 140% increase from 2023 to 2024), and a further reduction in our cancelation rate to 7% (a 50% reduction from 2023 to 2024).

Table 1 presents the annual breakdown of scheduled versus completed simulations. The simulation run rate remained stable at 81% throughout the study period (through June 2024), with narrowing control limits on the P-chart (Fig. 2), indicating reduced variability and improved predictability. The C-chart (Fig. 3) showed special-cause variation, with a centerline shift from 3.25 to 6.22 simulations per month. Following the intervention period (July 2024–June 2025), the average simulations per month increased to 10.63, and the run rate rose to 94%.

### Secondary Outcome Measures

From July 1, 2023, to June 30, 2024, a total of 89 simulations occurred involving 1,265 staff. This number reflects instances of participation rather than unique individuals; staff who participated in multiple simulations were counted multiple times. Descriptions of the simulation encounter types can be found in Table 2, the frequency of these types is shown in Table 1, and participant roles and counts are shown in Table 3.

**Table 3.** Participants in the PICU Simulation Program During the 2023 Academic Year

Participant Type	Number
Respiratory therapists	106
Pharmacists	17
Patient care assistances	28
Nurses	643
Advanced practice providers	71
Attendings	70
Fellows	166
Residents	62
Learners from other departments	113
Total	1265

### Process Measure

The simulation team audited scenario setup and takedown times before and after interventions. In the 2021–2022 academic year (baseline), the average setup time was 190 minutes, and the average takedown time was 110 minutes. After all 5 PDSA cycles, these times decreased to 80 (58% reduction) and 40 minutes (64% reduction), respectively.

### Balancing Measures

There were no logged safety reports in the institutional safety reporting system that identified a PICU simulation as a direct or potential cause of an actual or potential safety event.

## DISCUSSION

In this quality improvement initiative, we substantially increased the overall frequency and reliability of a PICU-based simulation program by redesigning how simulations were organized, selected, and executed. Reliability, defined as the consistency with which scheduled simulations were delivered, improved once the team established clear, consensus-driven criteria for when and how simulations should run, enabling rapid and consistent deployment of sessions and reducing cancelations. Efficiency, defined as reducing the time, labor, and operational friction required to plan, set up, run, and break down simulations, also improved, yielding marked reductions in setup and takedown time. Together, these gains were achieved without reported safety events or disruptions to clinical care, demonstrating that adaptable, demand-responsive models of in situ simulation can function safely within a high-acuity ICU environment.

Our findings build on prior literature describing the challenges of sustaining in situ simulation in dynamic clinical environments. Previous studies have highlighted barriers such as staffing variability, workflow interruptions, and limited interdisciplinary engagement,^12,14^ often resulting in inconsistent delivery of simulation training. In adult ICUs^24^ and emergency department settings,^25^ recurrent in situ simulation has been shown to be valuable, but studies generally emphasize feasibility, latent safety threat identification, or team performance rather than increasing simulation volume or improving reliability and execution efficiency. Similarly, content-specific efforts, such as low-dose, high-frequency cardiopulmonary resuscitation and neonatal resuscitation training,^26^ demonstrate the benefits of frequent, focused practice but do not address the system-level redesign required to scale simulation across diverse clinical scenarios. In contrast, our program combined standardized encounter types, real-time operational criteria, and predictive analytics to align simulation delivery with unit conditions, enabling expanded simulation volume, broader multidisciplinary participation, and more stable operational performance.

A key contributor to these improvements was the collaborative development of a simulation encounter decision tree that shifted the core question from “Can we run a simulation?” to “Which simulation format best fits current conditions?” This pivot strengthened adaptive capacity and aligned with safety science principles emphasizing systems that flex under fluctuating demand. Additionally, integrating scenario selection with real-time clinical risk indicators enabled more timely and relevant training, ensuring that simulation topics mirrored the evolving needs of the unit. Organizational changes, such as including centralized scenario kits (preprepared equipment associated with content-specific simulation), digital templates, and expanded interprofessional support—further reduced friction points that had previously limited consistency and scalability.

Diversifying our in situ simulation offerings meaningfully expanded opportunities for staff training. Adding Just-in-Time and Rolling Refresher formats provided brief, focused sessions that supplemented traditional high-fidelity, interdisciplinary simulations. Importantly, even when analyzing only the high-fidelity, unit-wide sessions that were directly comparable to the preintervention period, we observed a 109% increase in volume. These findings demonstrate that program growth reflected a true expansion in capacity rather than a reclassification of session types. Collectively, the structured changes to the simulation program increased the number of training touchpoints and broadened simulation access across clinical roles.

### Limitations

This study was conducted in a single high-acuity PICU, which may limit generalizability, though the clinical roles and workflows are similar to many academic ICUs. Our approach incorporated a predictive analytics tool not universally available, but similar methods could be applied using widely adopted early warning systems. Finally, we did not track specific failure modes leading to cancelations during the study period, limiting our ability to characterize remaining vulnerabilities.

### Next Steps

Future work will evaluate the comparative effectiveness of different simulation encounter types—such as Just-in-Time versus Core Team Training—on knowledge retention, communication, and clinical outcomes. Additional efforts to measure staff workload, cognitive burden, and engagement to ensure that efficiency gains do not mask unintended consequences represent key next steps. Finally, to aid with dissemination of this model to other clinical settings, the team will further quantify and qualify the key drivers of our program’s successful local expansion, including strong cross-disciplinary partnerships, alignment of topics with perceived clinical need, and deliberate investment in systems that support adaptive, high-frequency simulation delivery.

## CONCLUSIONS

This multiyear quality improvement initiative implemented a coordinated set of interventions to increase the adaptive capacity of a PICU-based simulation program, resulting in substantial gains in simulation frequency, reliability, and operational efficiency. Core components included standardized simulation encounter types, a consensus-driven decision tree to align simulation delivery with real-time operational conditions, enhanced interprofessional collaboration, integration of predictive analytics to guide scenario selection, and a comprehensive overhaul of simulation organization and resource management. These changes collectively enabled rapid, context-responsive deployment of simulation while reducing cancelations and maintaining safety within a high-acuity ICU environment.

The strategies described in this study offer a practical and scalable framework for other clinical settings seeking to build or expand in situ simulation capacity, particularly in environments where clinical demand and situational acuity fluctuate. By embedding structured adaptability, multidisciplinary input, and efficient resource use into simulation program design, institutions may strengthen both educational effectiveness and operational resilience.

## ACKNOWLEDGMENTS

The authors thank the staff of the CCHMC PICU for their ongoing support and dedication to training and improvement, as well as the CCHMC Center for Simulation and Research and the PICU Nursing Simulation Council for their invaluable contributions to this initiative.

## Supplementary Material


