# Supplementary material for: Building an Adaptable Pediatric Intensive Care Unit Simulation Portfolio: Advancing Efficiency, Flexibility, and Team-based Training
Source: Pediatr Qual Saf. 2025 Dec 23;10(6):e864. doi: 10.1097/pq9.0000000000000864 (PMC13169142; doi:10.1097/pq9.0000000000000864)
Supplement: Supplementary file 1 [file pqs-10-e864-s001.pdf]

Supplemental Table 1: PDSA cycle mapped to the SEIPS work-system elements

SEIPS ELEMENT	PDSA CYCLE(S) INVOLVED	KEY INTERVENTION(S) INTRODUCED	IMPROVEMENTS
PEOPLE	**Cycle 1 – Interprofessional Collaboration**	• Hired 0.5 FTE nurse-educator<br>• Cross-trained RT/RN/MD volunteers in rapid setup & debrief	Prep duties spread across a larger, trained pool → per-facilitator prep time ↓ 58 %
TASKS / WORKFLOW	**Cycle 2 – Standardize encounter types**<br>**Cycle 3 – Decision tree**<br>**Cycle 4 – Predictive-analytics scenario pick**	• Three clearly scoped formats (Core, JIT, Rolling Refreshers)<br>• Real-time decision algorithm chooses the *right* format for current acuity<br>• Warning-Tool triggers keep scenarios clinically relevant	Fewer late cancellations; sessions sized to unit load; learners perceive high relevance, so they willingly engage
TOOLS / TECHNOLOGY	**Cycle 5 – Resource Overhaul**<br>**Cycle 4 – Warning-Tool feed**	• Centralized closet + modular scenario kits + digital template vault<br>• Direct feed from PICU Warning Tool into scenario-selection huddle	Setup and takedown time decrease; “hunt-and-gather” stress eliminated; decision making data-driven, not guesswork
PHYSICAL SPACE	**Cycle 3 – Decision tree**	• Objective “no-go” criteria (census, staffing, >2 resuscitations) applied in twice-daily huddles to protect patient rooms	Same rooms, but conflicts now anticipated and avoided → no wasted effort on sessions that would be aborted
ORGANIZATION / POLICY	**Cycle 3 – Decision tree**<br>**Cycle 1 – Interprofessional Collaboration and Engagement**	• Leadership-approved algorithm embeds simulations into daily operations<br>• Interprofessional council stewards continuous process refinement	Unit views simulation as part of normal workflow, not an add-on; psychological resistance drops

SEIPS 2.0 elements: People, Tasks, Tools/Technology, Physical Environment, Organization/Policies.
