# Supplementary figures and images for: Building an Adaptable Pediatric Intensive Care Unit Simulation Portfolio: Advancing Efficiency, Flexibility, and Team-based Training
Source: Pediatr Qual Saf. 2025 Dec 23;10(6):e864. doi: 10.1097/pq9.0000000000000864 (PMC13169142; doi:10.1097/pq9.0000000000000864)

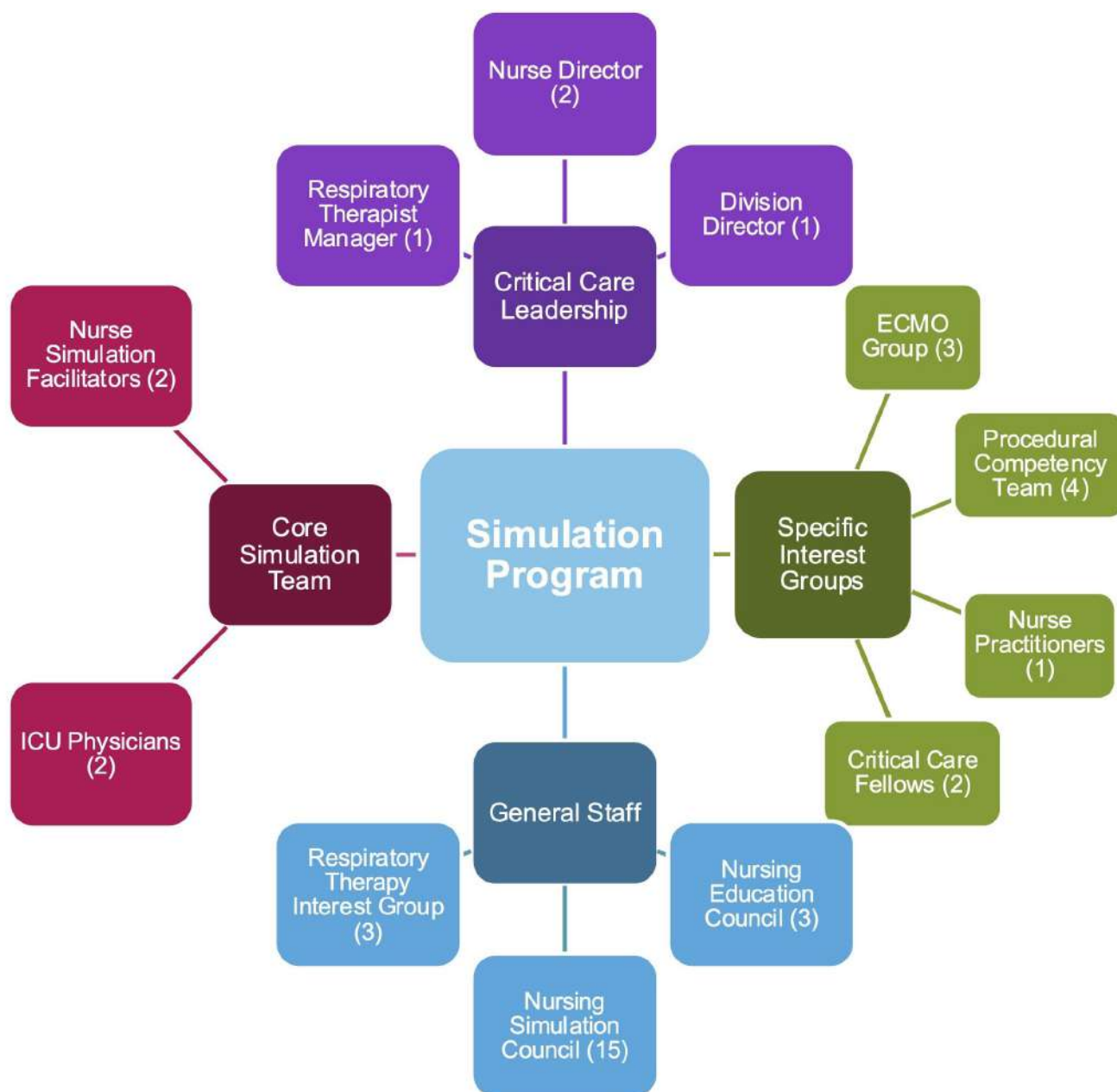

Supplement: Supplementary file 2 [file pqs-10-e864-s002.pdf]
