# Supplementary material for: Building an Adaptable Pediatric Intensive Care Unit Simulation Portfolio: Advancing Efficiency, Flexibility, and Team-based Training
Source: Pediatr Qual Saf. 2025 Dec 23;10(6):e864. doi: 10.1097/pq9.0000000000000864 (PMC13169142; doi:10.1097/pq9.0000000000000864)
Supplement: Supplementary file 3 [file pqs-10-e864-s003.pdf]

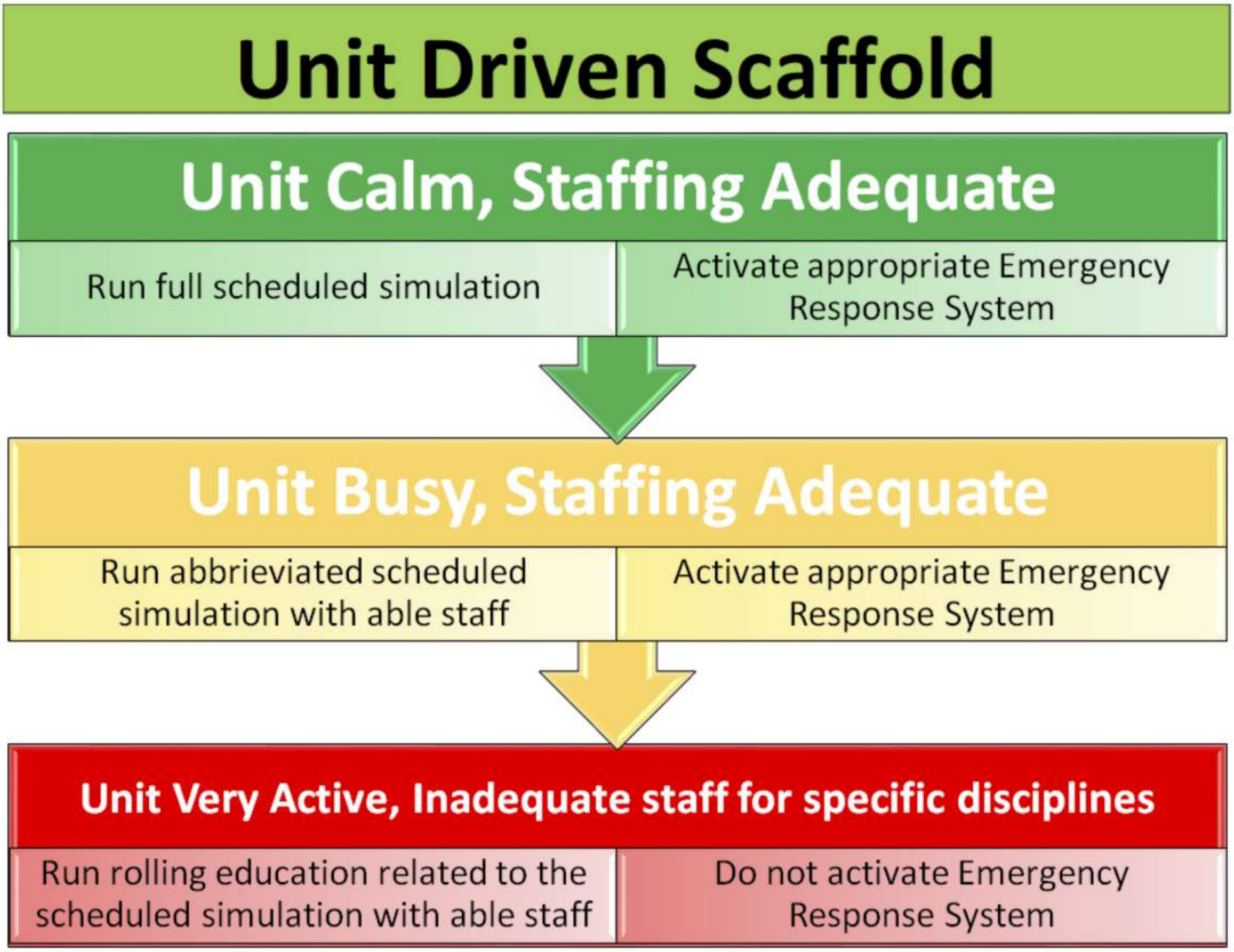
Unit Driven Scaffold
Unit Calm, Staffing Adequate
Run full scheduled simulation
Activate appropriate Emergency Response System
Unit Busy, Staffing Adequate
Run abbrieviated scheduled simulation with able staff
Activate appropriate Emergency Response System
Unit Very Active, Inadequate staff for specific disciplines
Run rolling education related to the scheduled simulation with able staff
Do not activate Emergency Response System
