# Supplementary material for: Building an Adaptable Pediatric Intensive Care Unit Simulation Portfolio: Advancing Efficiency, Flexibility, and Team-based Training
Source: Pediatr Qual Saf. 2025 Dec 23;10(6):e864. doi: 10.1097/pq9.0000000000000864 (PMC13169142; doi:10.1097/pq9.0000000000000864)
Supplement: Supplementary file 4 [file pqs-10-e864-s004.pdf]

Infant/TORY
(3-15 kg)
Kit A1

Inventory List

Resp. Supply Bag

- ❑ ½ L Mapleson
- ❑ Infant Mask
- ❑ BBG
- ❑ 3.0 Cuffed ETT
- ❑ Stylet
- ❑ 6 fr. In-line suction
- ❑ Christmas tree
- ❑ Oxy mask
- ❑ NC

Vitals Supply Bag

- ❑ Infant BP Cuff – cut
- ❑ Infant BP Cuff
- ❑ Cuff with Monitor ext. piece
- ❑ Baby Leads
- ❑ Pulse Ox low flow
- ❑ Pulse Ox

❑ 6 fr. Salum
❑ Feeding Tube
❑ Infant C-Collar
❑ Restraints

Toddler/Sim Baby
(15 - 18 kg)
Kit A2

Inventory List

Respiratory Supply Bag

- ❑ ½ L Mapleson
- ❑ Endtidal
- ❑ Cool mist
- ❑ Trach extension
- ❑ HME
- ❑ Child Mask
- ❑ 8 fr. In-line suction
- ❑ 5ml syringe

Trach Emergency Supply Bag

- ❑ 10 fr Cath
- ❑ 8 fr cath
- ❑ Hemostats
- ❑ Scissors
- ❑ Wedge
- ❑ Saline Bullets
- ❑ Lube
- ❑ 3 ml syringe
- ❑ Adult Trach ties
- ❑ Pediatric Trach ties

Vitals Supply Bag

- ❑ Infant BP Cuff
- ❑ Leads
- ❑ Pulse Ox low flow
- ❑ Pulse Ox

❑ Drainage Bag
❑ GT mickey
❑ Mepilex
❑ 4.0 Trach
❑ 4.5 Trach

Child/HAL
(19 - 40 kg)
Kit A2

Inventory List

Resp. Supply Bag

- ❑ 1 L Mapleson
- ❑ Child Mask
- ❑ 4.5 Cuffed ETT
- ❑ Stylet
- ❑ 8 fr. In-line suction
- ❑ Christmas tree
- ❑ Oxy mask
- ❑ NC
- ❑ Non-rebreather

Vitals Supply Bag

- ❑ Child BP Cuff – cut
- ❑ Child BP Cuff
- ❑ Cuff with Monitor ext. piece
- ❑ Leads
- ❑ Pulse Ox low flow
- ❑ Pulse Ox

❑ Salum
❑ Feeding Tube
❑ Pediatric C-Collar
❑ Restraints

Body Part Bag

- ❑ Chest tube insert
- ❑ Pneumothorax insert

Adult/Sim Man
(50 - 80 kg)
Kit A4

Inventory List

Intubation Supply Bag

- ❑ 1 L Mapleson
- ❑ Adult Mask
- ❑ 7.0 Cuffed ETT
- ❑ Stylet
- ❑ 14 fr. In-line suction

Oxygen Delivery Bag

- ❑ Christmas tree
- ❑ Oxy mask
- ❑ NC
- ❑ Non-rebreather

Vitals Supply Bag

- ❑ Small adult BP Cuff
- ❑ Leads
- ❑ Pulse Ox low flow
- ❑ Pulse Ox

❑ Salum
❑ Feeding Tube
❑ C-Collar
❑ Restraints
