# Supplementary material for: Building an Adaptable Pediatric Intensive Care Unit Simulation Portfolio: Advancing Efficiency, Flexibility, and Team-based Training
Source: Pediatr Qual Saf. 2025 Dec 23;10(6):e864. doi: 10.1097/pq9.0000000000000864 (PMC13169142; doi:10.1097/pq9.0000000000000864)
Supplement: Supplementary file 5 [file pqs-10-e864-s005.pdf]

Inventory List
Room Set Up

Intubation Supplies Bag
- ❑ 4.5 ETT
- ❑ Stylet
- ❑ In-line suction
- ❑ Towels
- ❑ Handle/Blades Bag
  - ❑ Lg. Laryngoscope Handle
  - ❑ Sm. Laryngoscope Handle
  - ❑ Miller 2
  - ❑ Phillips 1
  - ❑ Miller 0

Suction Tubing Bag
- ❑ Suction Tubing
- ❑ Suction Tubing
- ❑ Suction Tubing
- ❑ Suction Tubing
- ❑ Suction Tubing

Suction Caths/ Stethoscope Bag
- ❑ Stethoscope
- ❑ Clear Yankauer
- ❑ BBG
- ❑ 10 fr sx cath
- ❑ 10 fr sx cath
- ❑ 10 fr. Sx cath
- ❑ 14 fr. Sx cath
- ❑ 14 fr. Sx cath

Oxygen Delivery Bag
- ❑ Mapleson
- ❑ Nonrebreather
- ❑ NC
- ❑ Mask

- ❑ Suction Canister
- ❑ Suction Canister
- ❑ Suction Canister
- ❑ 1 Critical Measure
- ❑ PIV Setup
- ❑ PIV Setup
- ❑ Vent Calibration tubing
- ❑ A-line Kit
- ❑ iSTAT Machine
- ❑ Restraints
- ❑ Flashlight

Infant & Toddler Respiratory Supplies

Inventory List
- ☐ ½ L Mapleson
- ☐ Pediatric ambu
- ☐ 3 circuits
- ☐ Heater pot
- ☐ 2 filters

Child & Adult Respiratory Supplies

Inventory List
- ☐ 1L Mapleson
- ☐ End tidal
- ☐ BiPAP mask for SIM Man
- ☐ 2 Adult circuits
- ☐ Heater pot
- ☐ 2 filters

Miscellaneous Respiratory Supplies

Inventory List
- ☐ High Flow Circuit
- ☐ Continuous Neb
- ☐ Misty Neb
- ☐ Aeroneb
