# Supplementary material for: Building an Adaptable Pediatric Intensive Care Unit Simulation Portfolio: Advancing Efficiency, Flexibility, and Team-based Training
Source: Pediatr Qual Saf. 2025 Dec 23;10(6):e864. doi: 10.1097/pq9.0000000000000864 (PMC13169142; doi:10.1097/pq9.0000000000000864)
Supplement: Supplementary file 6 [file pqs-10-e864-s006.pdf]

JIT Specific Scenario

High risk Intubation

Patient Specifics

Manikin: Sim Man **Age:** 17 yrs **Weight:** 70 kg

History

Systematic-onset JIA, recurrent macrophage activation syndrome, IDA, HTN, first degree AV block, and MDD who presents as a transfer from ASH (Riley children's) for refractory MAS and possible Gamifant initiation (unavailable at OHS)

Signage

- [x] Watcher Sign
- [x] Airway Bundle
- [x] Code Sheet
- [ ] Sepsis Huddle
- [ ] TBI Care Plan
- [ ] Post Arrest Care Plan
- [x] Sim in progress sign
- [x] Insitu Cleanup Sheet
- [x] CME Code:
- [ ] Other:

Simulation Kits & Simulation Equipment

- [x] Specific Scenario Kit: Intubation Med Kit
- [x] Manikin Kit: Sim Man
- [x] Room Set-up Kit (Includes 2 PIV's & Aline)
- [x] Extra Access: PICC
- [x] Airway Cart
- [x] Training Crash Cart
- [x] Zoll & Pads
- [x] Patient Bed:
- [x] Pumps & Poles:
- [x] Vent
- [x] Other: Red tackle box

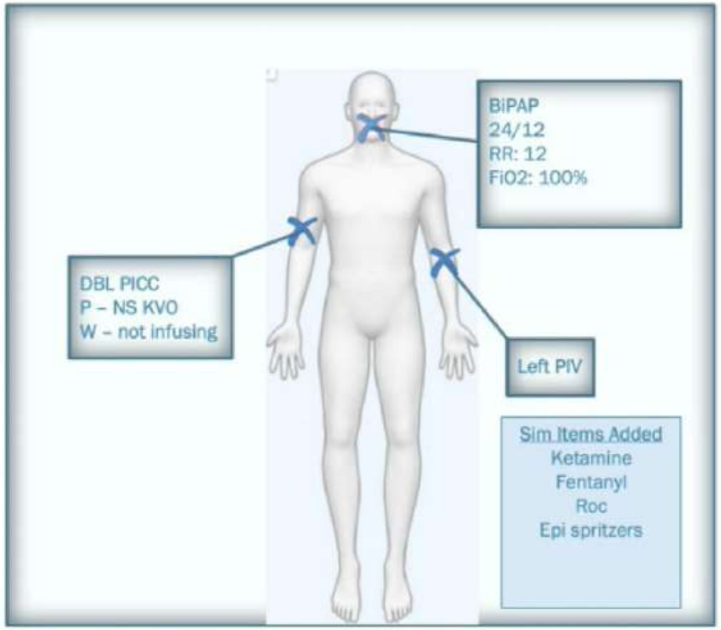

Vitals → Trend over 1min

HR: 130	→	PEA
RR: 30	→	PEA after Roc
02: 93	→	
ETCO2:	→	
BP: 145/95	→	60/40
Temp:		
Other:		

Manikin State/Labs/Imaging

- groans to sternal rub
- blink state → closed

Watcher Plan

High Risk Intubation
- Intubate pt
- Apneic Oxygenation

Patient has cardiac arrest?

Yes →

Team will recognize pt. has no pulse

- [x] Begin high quality CPR
- [x] Determine correct PALS algorithm
- [x] Give appropriate code meds
- [x] Review H's & T's
- [x] Other: Bring Storz to bedside, use RSI for intubation

No →

Team will recognize patient is peri-arrest. Team will initiate specific interventions:

- [ ]
- [ ]
- [ ]
- [ ]
- [ ]

iSTAT Results

PH:
PCO2:
BE:
HCT:
Hgb:
NA:
K:
Ical:
Gluc:
LA:

Desired Process? — No → **PAUSE REDIRECT & RESTART**

Pt with ROSC

Final Vitals	Manikin State
HR: 70	
RR: Assisted	
02: 88%	
ETCO2: 35	
BP: 100/70	
Temp:	
Other:	

Post Recovery State

Final Vitals	Manikin State
HR	
RR:	
02:	
ETCO2:	
BP:	
Temp:	
Other:	

Post Arrest or Post Recovery Goals:

- Go over post arrest goals for this patient, using care plan

END SCENARIO
